# Molecular degree of perturbation of plasma inflammatory markers associated with tuberculosis reveals distinct disease profiles between Indian and Chinese populations

**DOI:** 10.1038/s41598-019-44513-8

**Published:** 2019-05-29

**Authors:** Deivide Oliveira-de-Souza, Caian L. Vinhaes, Maria B. Arriaga, Nathella Pavan Kumar, Juan M. Cubillos-Angulo, Ruiru Shi, Wang Wei, Xing Yuan, Guolong Zhang, Ying Cai, Clifton E. Barry, Laura E. Via, Alan Sher, Subash Babu, Katrin D. Mayer-Barber, Helder I. Nakaya, Kiyoshi F. Fukutani, Bruno B. Andrade

**Affiliations:** 1 Instituto Gonçalo Moniz, Fundação Oswaldo Cruz, Salvador, 40296-710 Brazil; 2 Multinational Organization Network Sponsoring Translational and Epidemiological Research (MONSTER) Initiative, Fundação José Silveira, Salvador, 40210-320 Brazil; 3 Curso de Medicina, Faculdade de Tecnologia e Ciências (FTC), Salvador, 40290-150 Brazil; 4 National Institutes of Health- National Institute for Research in Tuberculosis, International Center for Excellence in Research, Chennai, 600031 India; 5 Henan Chest Hospital, Zhengzhou, 450000 China; 6 Sino-US International Research Center for Tuberculosis, and Henan Public Health Center, Zhengzhou, 450000 China; 7 Laboratory of Clinical Immunology and Microbiology, NIAID, NIH, Bethesda, 20892 USA; 8 Laboratory of Parasitic Diseases, NIAID, NIH, Bethesda, 20892 USA; 9 Department of Pathophysiology and Toxicology, School of Pharmaceutical Sciences, University of São Paulo, São Paulo, 05508 Brazil; 10 Division of Infectious Diseases, Department of Medicine, Vanderbilt University School of Medicine, Nashville, TN 37232 USA; 11 Universidade Salvador (UNIFACS), Laureate Universities, Salvador, 41720-200 Brazil

**Keywords:** Infection, Interleukins

## Abstract

Tuberculosis (TB) is a chronic inflammatory disease caused by *Mycobacterium tuberculosis* infection which causes tremendous morbidity and mortality worldwide. Clinical presentation of TB patients is very diverse and disease heterogeneity is associated with changes in biomarker signatures. Here, we compared at the molecular level the extent of individual inflammatory perturbation of plasma protein and lipid mediators associated with TB in patients in China versus India. We performed a cross-sectional study analyzing the overall degree of inflammatory perturbation in treatment-naïve pulmonary TB patients and uninfected individuals from India (TB: n = 97, healthy: n = 20) and China (TB: n = 100, healthy: n = 11). We employed the molecular degree of perturbation (MDP) adapted to plasma biomarkers to examine the overall changes in inflammation between these countries. *M. tuberculosis* infection caused a significant degree of molecular perturbation in patients from both countries, with higher perturbation detected in India. Interestingly, there were differences in biomarker perturbation patterns and the overall degree of inflammation. Patients with severe TB exhibited increased MDP values and Indian patients with this condition exhibited even higher degree of perturbation compared to Chinese patients. Network analyses identified IFN-α, IFN-β, IL-1RI and TNF-α as combined biomarkers that account for the overall molecular perturbation in the entire study population. Our results delineate the magnitude of the systemic inflammatory perturbation in pulmonary TB and reveal qualitative changes in inflammatory profiles between two countries with high disease prevalence.

## Introduction

Tuberculosis (TB) is now the leading cause of mortality worldwide due to a single infectious agent^1^. In addition, *Mycobacterium tuberculosis* (Mtb) is widely disseminated geographically, with up to 23% of the world’s population exposed to the bacterium. India and China are in the top five group countries having the largest number of cases in 2016, 56% of the total, according to the World Health Organization (WHO). With such a high burden of disease, associated with the socioeconomics conditions, the treatment and control of new cases is only partially effective in both countries, contributing to the appearance of multi-drug resistant strains, that represent 25% of cases in China and the emergence of totally resistant strains in India^2^. Furthermore, despite tremendous efforts from both private and governmental initiatives to reduce TB burden, deaths attributed to this disease remain high in both countries. Noteworthy, India presents substantially higher mortality rates than China in both HIV unexposed and exposed populations^1^. The major determinants of such high burden and mortality are still largely unknown.

Numerous factors are known to influence TB risk and disease presentation, including genetic^3^, epigenetic^4^, environmental^5^, nutritional^6^ and socioeconomic^7^ characteristics. India and China are very distinct with regard to several of these determinants, which could contribute to the diversity in TB clinical presentation and likely in clinical prognosis such as response to treatment. Whether such differences also result in divergences in immune and inflammatory responses against Mtb is not completely understood.

Here, we applied an adaptation of the molecular degree of perturbation (MDP) to previously published data on plasma concentrations of a defined panel of protein and lipid mediator biomarkers from China^8^ and from India^9^. This statistical approach was employed to determine possible differences in the inflammatory stress in pulmonary tuberculosis (PTB) between the two countries when the biomarker measurements were performed in different laboratory facilities. The concept which gives basis for calculation of the MDP scores is inspired by the Molecular Distance to Health^10^ and infers a degree of difference in expression levels of biomarkers between cases and controls (healthy uninfected controls), such differences were compared between countries. In addition, we determined the distance to health in mild TB (acid-fast bacilli [AFB] negative and unilateral lung lesion) and severe TB (AFB positive and bilateral lung lesion) and the interference of the imbalance caused by Mtb on the inflammatory profile in both countries. The results of this analysis have important implications for the design of more uniform measures for the diagnosis and subsequent treatment of TB.

## Materials and Methods

### Ethics statement

All clinical investigations were conducted according to the principles expressed in the Declaration of Helsinki. Written informed consent was obtained from all participants or their legally responsible guardians before enrolling into the sub-studies. The South Indian study was approved by the Institutional Review Board of the National Institute for Research in Tuberculosis (NIRT; protocol numbers NCT01154959 and NCT00342017). The Chinese study is registered on the platform ClinicalTrials.gov (NCT01071603) and has been approved and reviewed by the Institutional Review Board (IRB) from Henan Chest Hospital (HCH), China, and US National Institute for Allergy and Infectious Disease (NIAID), National Institutes of Health (NIH), Bethesda, Maryland.

### Study design and participants in China

The original clinical protocol, from which the biomarker study was derived, was performed in Zhengzhou, China, between 2010 and 2012^8^ with inclusion criteria of persons with signs and symptoms indicative of active TB, who were HIV-unexposed and administered less than two weeks of anti-tubercular treatment (ATT) and exclusion criteria of HIV infection, diabetes, cancer and other lung diseases identified at the first clinical visit and reported resistance to anti-TB drugs. In the present study on biomarkers, only treatment naïve individuals were included to reduce variability. Plasma samples were collected from 100 patients with active PTB and 11 healthy controls (HC). Six patients with PTB were excluded due to lack of sample available for the laboratory assays. All the enrolled subjects underwent a chest Computer Tomography (CT) scan, provided three sputum samples for AFB smear and culture by both the BACTEC MGIT 960 system (Becton, Dickenson and Company) and Lowenstein–Jensen medium (Chuang Xin Company; Hangzhou, China). The HCH radiology department provided a scored assessment of the chest CTs that included locations of disease by lobe, type of abnormalities and number of cavities for each CT. The sputum smears were prepared by the Zeil–Neilsen method using 1% carbolfuchsine and scored using the International Union Against Tuberculosis and Lung Disease (IUATLD) scale. HC participants presented in this analysis lacked radiologic and microbiological signs of active *M. tuberculosis* infection, had no pulmonary symptoms of tuberculosis and had a negative result in the Quantiferon Gold in-the-tube test.

### Study design and participants in India

Active TB cases were recruited at the Government Stanley Medical Hospital, at TB clinics supported by the National Institute for Research in Tuberculosis in Chennai, India. The diagnosis of PTB was based on sputum smear and culture positivity. At the time of enrollment, all active TB cases had no record of prior TB disease or ATT. ATT was given to all patients with active TB using the directly observed treatment, short course (DOTS) strategy following the WHO guidelines^11^. Healthy donors were asymptomatic with normal chest X-rays, negative tuberculin skin test (TST) (indurations, 5 mm in diameter) and Quantiferon as well as negative sputum smear or culture results. All participants were BCG vaccinated and were HIV negative. Plasma samples were collected at the time of study enrollment from 97 patients with active PTB, and 20 healthy donors recruited in Chennai, India, as part of a TB study.

### Immunoassays

Assessment of biomarkers of inflammation and immune activation were measured using commercial ELISA kits (R&D Systems, Minneapolis, MN) and Flowcytomix Multiplex Arrays (eBioscience, San Diego, CA) according to the manufacturer’s instructions. Levels of prostaglandins, leukotrienes and lipoxins (PGE2, PGF2a, LXA4 and 15-EPI-LXA4) were assessed using EIA kits according to the manufacturer’s directions (Oxford Biomedical Research, Oxford, MI).

### Data analysis

Categorical data were presented as proportions and continuous data as medians and interquartile ranges (IQR). The Fisher’s exact test was used to compare categorical variables between study groups. Continuous variables were compared using the Mann-Whitney *U* test. P-values were adjusted for multiple measurements using Holm-Bonferroni’s method. A multivariable regression model using variables with univariate p-value ≤ 0.2 was performed to assess the odds ratios (OR) and 95% confidence intervals (CIs) of the associations with country of residence. Hierarchical cluster analyses (Ward’s method), with 100X bootstrap of z-score normalized data were employed to depict the overall expression profile of indicated biomarkers in the study groups. Dendograms represent Euclidean distance.

Profiles of correlations between biomarkers in different clinical groups were examined using network analysis of the Spearman correlation matrices (with 100X bootstrap). In indicated analyses, only correlations with Spearman rank coefficient (r) values > 0.7 or <−0.7 and p-value < 0.05 after adjustment were included in the network visualization. In such analyses, markers that exhibited similar correlation profiles were clustered based on a modularity^12^, which infers sub-networks inside the of the correlation network and depicted using Fruchterman Reingold (force-directed graph drawing)^13^.

Sparse canonical correlation analysis (CCA) modelling was employed to assess whether combinations of circulating biomarkers could discriminate between subgroups of patients. The CCA model was chosen because many variables were studied. This model is able to perform dimensionality reduction for two co-dependent data sets (MDP biomarker profile and baseline characteristics profile, which were age and gender) simultaneously so that the discrimination of the clinical endpoints represents a combination of variables that are maximally correlated. Thus, trends of correlations between parameters in different clinical groups rather than their respective distribution within each group are the key components driving the discrimination outcome. In our CCA model, simplified and adapted from previously reported investigations of biomarkers for TB diagnosis^8,14^, linear regression graphs represent coefficients from different combinations of plasma factors and baseline characteristics. In the biomarker profile dataset, we included values of all the inflammatory marker variables. The diagnostic class prediction values obtained were calculated using receiver operator characteristics (ROC) curve analysis.

### Adaptation of the molecular degree of perturbation to examine plasma concentrations of biomarkers

The plasma measurements of both datasets were normalized equally with a log2 transformation and the batch effect within the different study datasets was corrected using Combat algorithm from SVA package^15^. The ComBat algorithm is a widely used method for adjusting batch effects in microarray and RNA-Seq data associated with technical variance effects. The molecular inflammatory perturbation is based on the  molecular degree of perturbation (MDP) method, which is an adaptation of the MDH described by Pankla *et al*.^10^. In the present study, instead of using gene expression values as in Prada-Medina *et al*.^16^ we inputted plasma concentrations of a defined set of biomarkers pre-selected based on previously published studies from our group which investigated TB pathogenesis^8,17^. Thus, herein, the average plasma concentration levels and standard deviation of a baseline reference group (healthy uninfected controls) were calculated for each biomarker. The MDP score of an individual biomarker was defined by taking the differences in concentration levels from the average of the biomarker in reference group divided by the reference standard deviation. Essentially, the MDP score represents the differences by number of standard deviations from the healthy control group. The formulas used to calculate MDP in the present study are shown below:

$$\begin{array}{ccc}Molecular\,inflammatory\,perturbation & = & \frac{{{\rm{x}}}_{{\rm{i}}}-{\bar{{\rm{x}}}}_{({\rm{r}}{\rm{e}}{\rm{f}}{\rm{e}}{\rm{r}}{\rm{e}}{\rm{n}}{\rm{c}}{\rm{e}})}}{{\sigma }_{({\rm{r}}{\rm{e}}{\rm{f}}{\rm{e}}{\rm{r}}{\rm{e}}{\rm{n}}{\rm{c}}{\rm{e}})}}\\ \sigma  & = & \frac{{\sum }_{(i=1)}^{n}{({{\rm{x}}}_{{\rm{i}}}-\bar{{\rm{x}}})}^{2}}{(n-1)}\end{array}$$

n = Number of data points

x_i_ = Each of the value of data

$\bar{{\rm{x}}}$ = Mean of the data points

σ = Standard deviation

In this study we applied the MDP scoring system using data on 15 biomarkers measured from two distinct countries of patients with TB and healthy uninfected controls. The MDP transformation was used as an approach to normalize data cross experiments resulting in datasets with markers distributed in a similar scale.

The MDP was filtered by the absolute MDP scores below 2 module and sum of all deviations accumulated MDP. To identify which sample was “perturbed”, we calculated the cutoff of the average MDP scores plus 2 standard deviations of the reference group and all values above this threshold was considered “perturbed”.

## Results

### Characteristics of study participants

In the Chinese study, plasma samples originated from persons with culture-confirmed pulmonary TB, who were HIV-uninfected and who had not been treated with anti-tubercular chemotherapy or community uninfected controls. These subjects were enrolled into a prospective clinical protocol to assess response to chemotherapy (NCT01071603) conducted at the Henan Chest Hospital (HCH) in Zhengzhou, China from 2010 to 2012, as described previously^8,18,19^. The present study was nested with this protocol and performed a cross-sectional analysis of treatment-naïve participant at the time of enrollment (baseline). Patients with PTB were similar to those presenting with uninfected controls with regard to age (median [IQR] in years: 27 [23–44.7] vs. 35 [23–40], respectively; P = 0.8020; Table S1) and gender (P = 0.5267; Table S1), with higher frequency of male individuals (61.7% vs. 45.4%, respectively).

In India, baseline plasma samples were collected from individuals with culture-confirmed pulmonary TB and who were HIV negative and had received no treatment for TB and had no record of prior TB disease recruited or uninfected healthy controls (NCT01154959 and NCT00342017) recruited at the National Institute for Research in Tuberculosis, Chennai, India. Individuals with PTB were similar with regard to sex but older than healthy controls (median [IQR] in years: 38 [28–47] vs. 28.5 [26–35], respectively; P = 0.0119; Table S2).

In addition, PTB individuals from India had a similar frequency of males as those from China (Table S3). However, Indian PTB patients were older than Chinese. Disease presentation was also distinct, with Indian patients exhibiting higher AFB smear grades whereas Chinese patients more frequently had bilateral lung lesions (Table S3).

### Increases in molecular degree perturbation of plasma cytokines and lipid mediators in pulmonary tuberculosis

Plasma levels of 15 cytokines and lipid mediators were compared between PTB patients and uninfected healthy controls in studies from China and India, separately (Table S4 and S5). In China, PTB was associated with higher levels of PGF2α and PGE2 than that measured in healthy controls (Table S4). The other parameters were lower in active PTB, except for IFN-β, IL-10, LXA4 and sIL-1RI and sIL-1RII, levels of which were not different between the study groups (Table S4). Compared to uninfected controls, Indian individuals with PTB exhibited higher levels of most parameters, except for IL-18 and PGF2α which were lower and IFN-β, IL-12p70, IL-10 and sIL-1RII, that did not differ (Table S5). These results suggested that there are differences in biomarker profiles in active PTB in geographically distinct regions. Similar conclusions have been published previously for persons living with HIV^20^.

**Figure 1:**
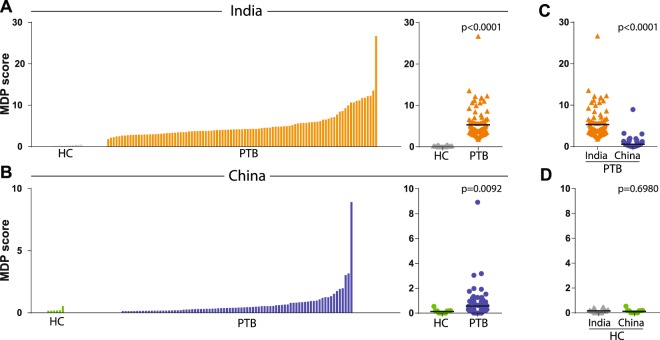
Pulmonary tuberculosis patients from both India and China exhibit substantial molecular degree of perturbation. (**A,B**) Left panels: Histograms show the single sample molecular degree of perturbation (MDP) score values relative to the healthy control group for each country (pulmonary TB: PTB, healthy controls: HC). MDP values were calculated as described in Methods. Right panels: Scatter plots of the summary data for each country are shown. MDP score values were compared between PTB patients (**C**) or healthy controls (**D**) from India or China. Lines in the scatter plots represent median values. Data were compared using the Mann–Whitney *U* test.

**Figure 2:**
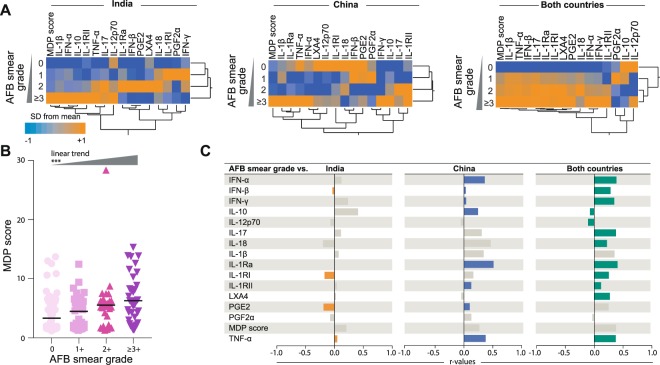
Association between mycobacterial loads in sputum smears and molecular perturbation of plasma biomarkers. (**A**) Hierarchical cluster analysis (Ward’s method with 100X bootstrap) using z-score normalized values of overall MDP score of individual MDP for each biomarker was employed to illustrate the overall expression profile in PTB patients stratified per AFB smear grade in India, China or all patients combined. Dendrograms represent Euclidean distance. **(B)** Scatterplots of overall MDP score values in all PTB patients (n = 191, 97 from India and 94 from China) stratified per AFB smear grade. Data were compared using the Kruskal-Wallis test with linear trend ad hoc test (***p < 0.0001). **(C)** Correlations between AFB smear grades and overall molecular degree of perturbation or perturbation of individuals markers in patients from India, China or altogether were tested using the Spearman rank test. Colored bars indicate significant correlations (p < 0.05).

In order to directly compare groups from the two different countries, we calculated the overall MDP score values. We found that active PTB was associated with a substantial increase in MDP scores compared to controls in both India (p < 0.0001, Fig. 1A) and China (p = 0.0092, Fig. 1B). Interestingly, PTB patients from India exhibited 3.7 times higher MDP values than those from China (p < 0.0001, Fig. 1C), whereas healthy control groups from both countries displayed indistinguishable values (p = 0.698, Fig. 1D). Because PTB patients from India and China were also different with regard to age, sex, high AFB smear grade (≥2+) and presence of bilateral lung lesions, it was important to test whether such differences account for the discrepancies in MDP values. To try to answer this question, we stratified the patients from both countries according to these characteristics (Table S6). Hierarchical cluster analysis was performed with the z-score normalized values of the overall MDP score as well as of that for each biomarker in patients presenting with different mycobacterial loads in sputum smears. This approach revealed that the highest levels of overall MDP scores were found in the group of TB patients exhibiting the highest AFB smear grades, in both countries (Fig. 2A). Indeed, linear trend analysis indicated that MDP score values gradually increased following augmentation of mycobacterial loads in sputum (p < 0.0001; Fig. 2B).

When individual MDP values for each marker was examined, we observed a highly heterogeneous distribution among the different subgroups of patients stratified by AFB smear grades (Fig. 2A). AFB smear grades were statistically correlated with values of different markers in India and China (Fig. 2C). In India, AFB grades were inversely correlated with IFN-β, sIL-1RI, PGE2 and directly associated with TNF-α. In Chinese patients, mycobacterial loads in sputum positively correlated with INF-α, IFN-β, IFN-γ, IL-10, IL-1Ra, sIL-1RII, PGE2 and TNF-α (Fig. 2). The AFB smear grade values did not correlate with MDP score values in patients from either India or China using Spearman test (Fig. 2), probably due to low dynamic range of data distribution. Of note, a multivariate logistic regression model demonstrated that 1 log increases in MDP score values were associated with PTB in India vs. in China (unadjusted OR: 2.06, 95%CI: 1.72–2.47, p < 0.001; adjusted OR: 6.23, 95% CI: 3.01–12.89, p < 0.001) independent on age, sex, high AFB smear grade (≥2+) and the presence of bilateral lung lesions (Fig. S1).

### Plasma markers driving the overall molecular degree of perturbation in pulmonary tuberculosis are distinct in patients from India versus China

**Figure 3:**
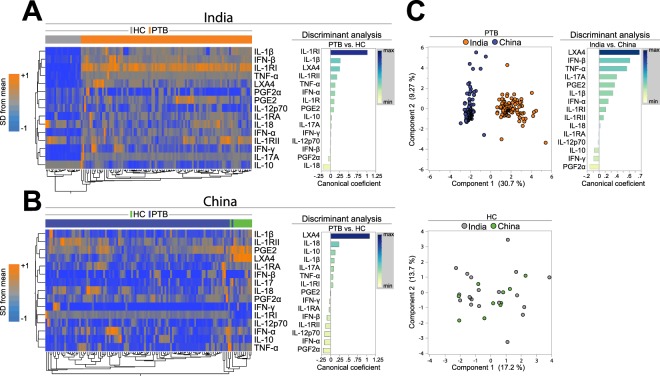
Plasma biomarkers driving the overall molecular degree of perturbation in pulmonary tuberculosis are distinct between patients from India and China. (**A,B**) Left panels: Unsupervised two-way hierarchical cluster analyses (Wards method with 100x bootstrap) using the MDP values for each individual markers measured in plasma from patients from both countries were employed to test if simultaneous assessment of such markers could group PTB separately from healthy individuals. Dendrograms represent Euclidean distance. Right panels: A discriminant analysis model based on canonical correlation analyses was used to identify the markers which are driving the discrimination between the study groups. (**C**) Left panels: A principal component model was used to illustrate segregation between PTB patients, but not healthy controls (HC) from India or China. Right panel: A discriminant analysis model based on canonical correlation analyses was used to identify the markers which are driving the discrimination between patients from India or China. In the discriminant analyses, biomarkers with canonical coefficient scores above 0.2 and below −0.2 were considered most influential in the models. Number of patients per group: India HC: n = 20, India PTB: n = 97, China HC: n = 11, China PTB: n = 100.

When molecular degree perturbation (MDP) for each individual plasma cytokine or lipid mediator were analyzed using unsupervised hierarchical clustering, we found that the overall expression profile was also very distinct between PTB patients and uninfected controls in both countries (Fig. 3A,B, left panels). Univariate analyses confirmed the differences in several markers (Fig. S2). Additional analysis revealed that frequency of individuals with molecular perturbation of IL-18, IFN-α, TNF-α, sIL-1RI, IL-17 and IL-1β was higher in PTB patients than in controls whereas only sIL-1RI was more often molecularly perturbed in Chinese TB patients (Fig. S3). Further investigation utilizing a discriminant model based on canonical correlation analysis confirmed that the combination of the plasma markers exhibited high accuracy in identifying PTB both in India (ROC AUC: 1.0, p < 0.0001) and China (ROC AUC: 0.98, p < 0.0001). Moreover, the canonical model indicated that the markers that contributed the most for the discrimination in India were IL-1RI, IL-1β, LXA4, IL-1RII and IL-18 whereas the markers identified in China were LXA4, IL-18, PGF2α, IFN-α, IL-12p70 and IL-1RII (Fig. 3A,B, right panels). Interestingly, molecular degree of perturbation of 3 out of 5 markers which were relevant to identify PTB patients in India (LXA4, IL-1RII and IL-18) were also part of the TB signature observed in China, revealing a degree of similarity. Nevertheless, when values from all the markers were considered simultaneously for the PTB patients, we found that the MDP score profiles were very distinct between India and China (Fig. 3C). In contrast these profiles were not different between uninfected controls from either country (Fig. 3C). The top 5 markers driving the discrimination between the PTB groups from different countries were LXA4, IFN-β, TNF-α, IL-17A and PGE2 (Fig. 3C, right panel). A logistic regression model confirmed that increases in MDP values of IFN-α and IFN-γ were independently associated with Indian subjects whereas increases in IL-10 MDP values were associated with Chinese patients (Fig. S4). These results suggest that PTB drives changes in the molecular degree of perturbation that are influenced by geographic region.

### Systemic inflammatory profile of severe TB

**Figure 4:**
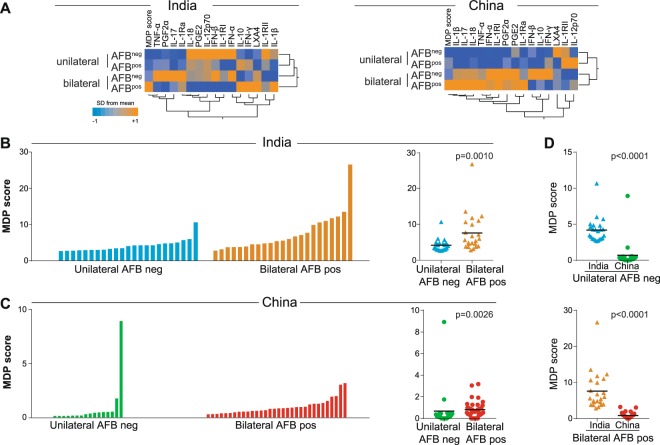
Indian patients with PTB exhibit increased molecular degree of perturbation than Chinese independent of disease extension status. PTB patients from both countries were stratified based on lung disease extension using smear acid-fast bacilli (AFB) status (negative or positive) and radiographic distribution of lung lesions (unilateral or bilateral). (**A**) Hierarchical cluster analysis (Ward’s method with 100X bootstrap) was employed to illustrate the overall expression profile of the biomarkers in PTB patients stratified per AFB smear status and lung disease extension. Dendrograms represent Euclidean distance. (**B,C**) Left panels: Histograms show the single sample molecular degree of perturbation (MDP) score values in the subgroups of PTB patients. Right panels: Scatter plots of the summary data for each country are shown. (**D**) MDP score values were also compared between the subgroups of PTB patients between India and China. Lines in the scatter plots represent median values. Data were compared using the Mann–Whitney *U* test.

In addition to mycobacterial loads in sputum, radiographic disease extension has been used by our group and others as a potential parameter of TB disease severity^16,21–23^. We have previously compared the inflammatory profile of active TB patients presenting with either mild (AFB negative in sputum smears and unilateral lung lesions) or severe (AFB positive and bilateral lung lesions) separately from India and China^8^. In the present study, we compared the inflammatory perturbation within each country and between them (Fig. 4). The highest MDP values were found in patients presenting severe TB when compared with those with mild disease in both countries. (Fig. 4A–C). Intriguingly, patients with both mild and severe disease from India showed higher values of MDP when compared to those from China (Fig. 4D). The individual values of MDP per marker highlighted a heterogeneous but distinct profile between the study groups (Fig. S5). These results suggest that molecular degree of perturbation of lipid mediators and soluble inflammatory proteins is an underlying feature of the observed changes in inflammatory milieu of PTB. Importantly, this analysis emphasizes that individuals from India show increased molecular perturbation when compared with Chinese patients independent of the disease severity.

### Network analysis of molecular degree of perturbation

**Figure 5:**
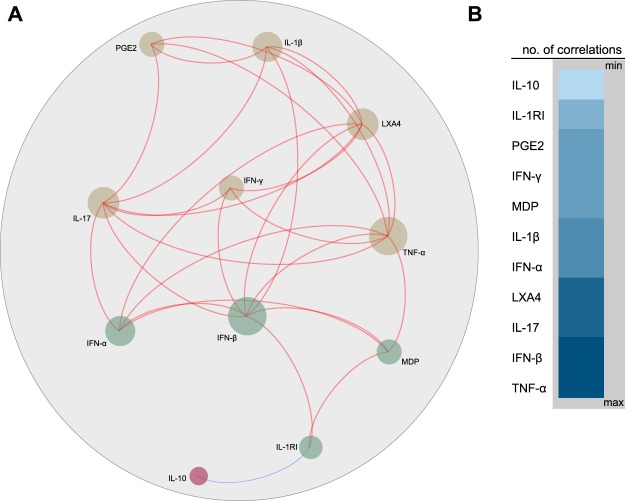
Network analysis of the molecular degree of perturbation score values in all participants from India and China. A Spearman correlation matrix including the molecular degree of perturbation values for each biomarker as well as the single sample molecular degree of perturbation was built. (**A**) Network illustrates strong Spearman correlations (p < 0.001; Spearman rank value > 0.7 or <−0.7). Markers were clustered based on a similarity index of the correlation profiles using a modularity algorithm and depicted with Fruchterman Reingold (force-directed graph drawing). Using this approach, three main nodes were detected. Only markers which had strong correlations were plotted to reduce visual pollution. Red lines represent positive while blue lines indicate negative correlations. **(B)** Node analysis was used to illustrate the number of strong correlations per marker. Markers were grouped according to the number of connections from minimum to maximum numbers detected.

To further explore nuances between the molecular perturbation of individual markers as well as their direct effect on overall MDP values, we employed network analysis based on Spearman correlation matrices. This analysis allowed to merge data from PTB and healthy controls from both countries. Using this approach, we observed that most of the significant correlations between the markers were positive, except for the association between IL-10 and sIL-1RI, which was negative (r: −0.72, p < 0.001). Furthermore, the markers exhibiting the highest numbers of significant correlations in the network were TNF-α, IFN-β, IL-17, LXA4 (Fig. 5). Of note, we also found that the degree of perturbation in TNF-α, IFN-α, IFN-β and sIL-1RI markers was directly associated with the overall molecular perturbation (Fig. 5). These findings highlight the specific molecular determinants driving the overall perturbation in PTB.

## Discussion

Multiple studies of TB biomarkers have been performed in a diverse range of settings, including different countries with genetically distinct individuals. Many of these studies employ distinct methodologies to measure the analytes and for this reason, even in scenarios in which clinical and microbiological definitions are harmonized, there is a significant challenge to compare results and perform analyses across countries. This issue potentially delays the validation of biomarkers and, maybe more importantly, the progress towards our understanding about nuances in TB pathogenesis in diverse populations. In order to circumvent this issue, multicentric studies perform measurements in a single or few laboratory facilities, mitigating inter-assay variability. In the present study, we have introduced a mathematical maneuver that employs standardization of biomarker data from TB patients over measurements from a healthy, uninfected control group (baseline reference) which results in a new metric that represents molecular distance to health^10^ or molecular degree of perturbation^16^. This type of approach has been used previously in the context of gene expression assays such as transcriptome analyses^16,24^. The results presented here expand the current knowledge in this field by demonstrating that molecular degree of perturbation can be used to compare soluble protein and lipid mediator biomarker profiles in plasma from TB patients with measurements performed at different laboratories in distinct countries.

A major observation of our study was that, although healthy uninfected controls from both India and China were undistinguishable in terms of MDP values, PTB patients exhibited very distinct values. Indian PTB patients were on average >3 times more molecularly perturbed than those from China. Although PTB patients from India had more frequent unilateral lung lesions and increased AFB smear grades than those from China, the association between increases of overall MDP score values and India was independent of such clinical and microbiological characteristics. This finding is intriguing and suggests that other factors may account for the differences in MDP. The impact of country of residence^20^, race and ethnicity^25^ on plasma biomarkers in the context of TB has been previously explored. Race and gender differences in inflammatory profile have also been studied in several other diseases^26–29^. It is likely that Indian and Chinese individuals respond differently in terms of immune response to Mtb infection. Indeed, additional analyses revealed that the biomarkers examined here, when considered combined, could cluster TB away from uninfected individuals in both countries, but were at the same time capable of discriminating TB patients from the two different countries. Hierarchical clustering, PCA and canonical discriminant analyses confirmed that healthy controls were very similar between India and China, whereas TB was associated with substantial discrepancies in the systemic inflammatory profile. Thus, the impact of ethnicity and/or country of residence on systemic inflammation was dependent on an environmental trigger, exemplified here by Mtb infection.

Of note, the discriminant analyses based on canonical correlations identified that the biomarkers likely responsible for the distinction between active TB and healthy controls were different between the countries. In India, sIL-1RI was the marker that contributed the most for this discrimination, with values significantly higher in active TB patients compared to uninfected controls. In China, the top discriminant marker was LXA4, with substantially lower levels detected in active TB versus controls. The most relevant markers driving the distinction of the inflammatory profiles between the countries were LXA4, IFN-β and TNF-α, all of which have been described to play relevant roles in TB pathogenesis^30^. LXA4 promotes necrosis of macrophages infected with Mtb by interfering in cell membrane repair^31,32^. In addition, we and others have shown that type I IFNs promote mycobacterial growth and unrestrained inflammation-driven tissue damage in mice and patients with more severe forms of TB^33^. The detrimental role of type I IFNs also involves inhibition of the IL-1 pathway which is required to induce host resistance against Mtb infection^8,34^. TNF-α is described as one of the most important cytokines in the protective immune responses against TB^35^. Loss of TNF signaling causes progression to active TB in Mtb infected individuals^35^, likely due to disruption of granulomas and accelerated intracellular mycobacterial growth^36^. Host genetic and/or epigenetic differences in expression of the markers described above as well as in other related immune pathways could be the underlying factors driving such discrepancies in different countries.

Overall, in transcriptome studies, MDP scores serve as a readout of the inflammatory activity^16^. In agreement with this idea, our results in protein quantification indicate that, in both India and China, PTB patients with bilateral lung lesions and presence of AFB in sputum smears exhibited higher MDP score values than those with unilateral lesions and negative smears. This finding is not surprising as progression of TB and increased mycobacterial loads drive major stress in the immune system^8,22^. Importantly, patients with PTB from India were more molecularly perturbed than those from China independent on the bacterial loads and/or radiographic extension of lung lesions. These results argue that country of origin may be a relevant driver of certain observed differences in pro-inflammatory biomarker expression in plasma of PTB patients.

One limitation of the present study was that we have not performed detailed genetic characterization of the Mtb isolates. The mycobacterial strains responsible for TB in India and China may differ. Studies have indicated that in India, the most prevalent Mtb lineage is the SIT26/cas1-Delhi^37^, whereas in China, Beijing is the most relevant strain^38,39^. Nevertheless, Mtb lineages are spread out around the world and Beijing strains are commonly found in India, for example^40^. Different Mtb isolates are well known to induce distinct immune activation and disease presentation in both experimental^17^ and clinical settings^41^. In fact, the Beijing lineage has been commonly associated with development of multi-drug resistant TB in several countries^39^. Hypervirulent isolates are known to cause significant lung necrosis and hematogenous dissemination^17^. It is possible that infection with distinct mycobacterial strains may be an important component underlying the differences in MDP score values between the countries evaluated. Should this hypothesis be correct, then it is likely that the majority of the Indian TB patients were infected by more virulent Mtb isolates. Perhaps more importantly, different countries may have unique endemic infectious agents other than Mtb, that could influence inflammatory responses and biomarker levels. In addition, the microbiome profile, which shows geographic variation and is highly influenced by diet and life-style^42^, could affect biomarker status. Thus, a combination of host and microbe genetic backgrounds may account for the systemic inflammatory changes described here. Future studies are needed to directly test this hypothesis. Another limitation of the present analysis was lack of control for air pollution^43^ as well as other behavioral and psychosocial factors, which may play a role in inducing changes in inflammatory responses as well. It is also possible that differences in time from TB disease onset to diagnosis differed may have resulted in distinctions in disease presentation between India and China, e.g. delayed diagnosis in India could have led to inclusion of patients with more advanced disease compared to that in China. We have not specifically tested this hypothesis and additional investigations are required. Finally, the data available for the researchers were not prospective, meaning that we did not have information on response to treatment or antibiotic resistance, which could have been different between the countries.

In summary, our data using the MDP metric suggest that systemic inflammation driven by Mtb infection may vary significantly between countries. Additional studies are needed to evaluate if and/or how the differences described here contribute to TB treatment response and prognosis in diverse populations worldwide.

## Supplementary information

Supplementary Tables and Figures

## Data Availability

The datasets generated during and/or analyzed during the current study are available from the corresponding author on reasonable request.
