# Supplementary material for: Molecular degree of perturbation of plasma inflammatory markers associated with tuberculosis reveals distinct disease profiles between Indian and Chinese populations
Source: Sci Rep. 2019 May 29;9:8002. doi: 10.1038/s41598-019-44513-8 (PMC6541651; doi:10.1038/s41598-019-44513-8)
Supplement: Supplementary file 1 — Supplementary Tables and Figures [file 41598_2019_44513_MOESM1_ESM.pdf]

Molecular degree of perturbation of plasma inflammatory markers associated with tuberculosis reveals distinct disease profiles between Indian and Chinese populations

Deivide O. Souza, Caian. L. Vinhaes, Marìa B. Arriaga, Nathella Pavan Kumar, Juan M. Cubillos-Angulo, Ruiru Shi, Wang Wei, Xing Yuan, Guolong Zhang, Ying Cai, Clifton E. Barry III, Laura E. Via, Alan Sher, Subash Babu, Katrin D. Mayer-Barber, Helder I. Nakaya, Kiyoshi F. Fukutani, Bruno B. Andrade.

Supplemental Tables and Figures

Table S1. Characteristics of the study participants from China

Characteristic	Healthy controls	PTB	P-value
N	11	94	
Age – y	35 (23-40)	27 (23-44.7)	0.8020
Male – no. (%)	5 (45.4)	58 (61.7)	0.5267
Positive AFB smear – no. (%)		50 (53.2)	
AFB smear grade – no. (%)			
0		58 (61.7)	
1+		15 (15.95)	
2+		8 (8.5)	
≥3+		13 (13.8)	
Bilateral Lesions – no. (%)		60 (63.8)	

Data represent medians and interquartile ranges (age) and frequencies (male gender). The Mann-Whitney test was used to compare continuous variables between the groups and the distributions of age while the Fisher's exact test was used to compare frequencies. AFB, acid-fast bacilli; CRP, C-reactive protein; PTB, active pulmonary TB; RBC, red blood cells; WBC, white blood cells. P-values in bold font are statistically significant.

Table S2. Characteristics of the study participants from India

Characteristic	Healthy controls	PTB	P-value
N	20	97	
Age – y	28.5 (26-35)	38 (28-47)	**0.0119**
Male – no. (%)	17 (85)	64 (65.9)	0.2689
Positive AFB smear – no. (%)		67 (69.1)	
AFB smear grade – no. (%)			
0		30 (30.9)	
1+		19 (19.6)	
2+		20 (20.6)	
≥3+		28 (28.9)	
Bilateral Lesions – no. (%)		30 (30.9)	

Data represent medians and interquartile ranges (age) and frequencies (male gender). The Mann-Whitney test was used to compare continuous variables between the groups and the distributions of age while the Fisher's exact test was used to compare frequencies. AFB, acid-fast bacilli; CRP, C-reactive protein; PTB, active pulmonary TB; RBC, red blood cells; WBC, white blood cells. P values in bold font are statistically significant.

Table S3. Characteristics of the study PTB patients from India and China

Characteristic	PTB India	PTB China	P-value
N	97	94	
Age – y	38 (28-47)	27 (23-44.7)	**0.0063**
Male – no. (%)	64 (65.9)	58 (61.7)	0.5507
Positive AFB smear – no. (%)	67 (69.1)	50 (53.2)	**0.0088**
AFB smear grade ≥2+ – no. (%)	48 (49.5)	21 (22.3)	**<0.0001**
Bilateral lesions – no. (%)	30 (30.9)	60 (63.8)	**0.0065**

Data represent medians and interquartile ranges (age) and frequencies (male gender). The Mann-Whitney test was used to compare continuous variables between the groups and the distributions of age while the Fisher's exact test was used to compare frequencies. AFB, acid-fast bacilli; CRP, C-reactive protein; PTB, active pulmonary TB; RBC, red blood cells; WBC, white blood cells. P values in bold font are statistically significant.

Table S4. Distribution of the plasma factors in the Chinese cohort at study entry

Parameter	Unit	Healthy controls	PTB	P-value
N		11	94	
IFN-β	pg/mL	0.1 (0.0-206.3)	2.2 (0.0-61.9)	>0.9999
IL-12p70	pg/mL	6.2 (4.8-12.3)	4.2 (3.3-7.8)	**0.0272**
IFN-γ	pg/mL	11.1 (3.4-15.8)	2.5 (1.9-4.4)	**0.001**
IL-17	pg/mL	74.0 (43.1-493.1)	17.1 (0.0-44.6)	**0.0006**
IL-10	pg/mL	215.0 (173.8-269.9)	163.6 (143.5-267.5)	0.089
IL-1RA	pg/mL	306.7 (282.3-371.9)	227.3 (196.0-311.7)	**0.0066**
IFN-α	pg/mL	125.4 (113.9-143.2)	98.9 (80.0-130.3)	**0.0443**
IL-18	pg/mL	905.1 (426.9-1204)	574.9 (388.1-992.6)	**<0.0001**
IL-1β	pg/mL	26.4 (20.7-86.0)	6.8 (0,0-16.7)	**<0.0001**
TNF-α	pg/mL	117.3 (76.9-268.2)	17.7 (0.0-75.3)	**0.0005**
PGE2	ng/mL	0.4 (0.3-0.6)	0.5 (0.4-0.7)	**0.0127**
LXA4	ng/mL	1.5 (1.2-1.7)	1.5 (1.3-1.8)	0.4135
PGF2α	ng/mL	0.05 (0.03-0.06)	0.06 (0.05-0.08)	**0.0038**
sIL-1R1	pg/mL	47.9 (42.9-56.3)	49.5 (46.2-55.0)	0.538
sIL-1R2	ng/mL	12.3 (9.0-13.6)	10.05 (8.8-12.4)	0.6986

Data represent medians and interquartile ranges. The Kruskal-Wallis test was used to compare the distributions of the plasma mediators between the study groups. P values in bold font are statistically significant

Table S5. Distribution of the plasma factors in the Indian cohort at study entry

Parameter	Unit	Healthy controls	PTB	P-value
N		20	97	
IFN-β	pg/mL	4.4 (2.2-5.5)	2.9 (1.3-7.1)	0.5936
IL-12p70	pg/mL	2.3 (1.6-3.3)	2.2 (0.9-5.3)	0.5183
IFN-γ	pg/mL	20.6 (15-19.48)	33.9 (29.81-35.32)	**<0.0001**
IL-17	pg/mL	16.3 (43.1-493.1)	27.1 (25.85-35.32)	**<0.0001**
IL-10	pg/mL	18.4 (16.43-20.85)	21.0 (15.93-29.05)	0.2740
IL-1RA	pg/mL	80.8 (62.55-98.18)	328.4 (229.2-527.1)	**<0.0001**
IFN-α	pg/mL	3.2 (1-4.3)	11.6 (7.9-17.3)	**<0.0001**
IL-18	pg/mL	933.6 (575.7-1217)	627.1 (367.9-823.8)	**0.0157**
IL-1β	pg/mL	2.8 (2.5-3.4)	15.9 (12.9-20.1)	**<0.0001**
TNF-α	pg/mL	12.5 (10.2-17.5)	21.3 (14.9-27.4)	**<0.0001**
PGE2	ng/mL	0.7 (0.5-1)	1.5 (0.9-2.1)	**<0.0001**
LXA4	ng/mL	1.2 (0.5-2)	7.4 (0.4-15.1)	**0.0018**
PGF2α	ng/mL	0.1 (0.1-0.2)	0.05 (0.02-0.2)	**0.0246**
sIL-1R1	pg/mL	163.5 (163.5-168.5)	296.5 (285.4-303)	**<0.0001**
sIL-1R2	ng/mL	14.7 (9.1-19.7)	13.4 (10.8-18.1)	0.8988

Data represent medians and interquartile ranges. The Kruskal-Wallis test was used to compare the distributions of the plasma mediators between the study groups. P-values in bold font are statistically significant.

Table S6. TB Patient stratified by AFB smear grade and lung lesions.

	India	China
N	97	94
AFB smear grade		
0	30	58
1+	19	11
2+	20	8
≥3+	28	13
Unilateral lung lesion	40	31
Bilateral lung lesion	30	55
Unilateral lung lesion + AFB neg (Mild)	23	22
Bilateral lung lesion + AFB neg	7	21
Unilateral lung lesion + AFB pos	17	09
Bilateral lung lesion + AFB pos (Severe)	23	34

Parameter	Model	Odds Ratio (95% CI)	p-value
**Male**	**unajusted**	2.00 (1.14-3.48)	0.02
	**ajusted**	1.06 (0.13- 8.84)	0.95
**Age**	**unajusted**	1.02 (0.95-1.03)	0.02
	**ajusted**	1.06 (0.98-1.15)	0.14
**MDP score**	**unajusted**	2.06 (1.72-2.47)	<0.01
	**ajusted**	6.23 (3.01-12.89)	<0.01
**Bilateral lung lesion**	**unajusted**	0.27 (0.12-0.56)	0.001
	**ajusted**	0.18 (0.02-1.27)	0.09
**AFB smear grade ≥ +2**	**unajusted**	3.68 (1.97-6.88)	<0.01
	**ajusted**	2.01 (0.30-13.54)	0.47

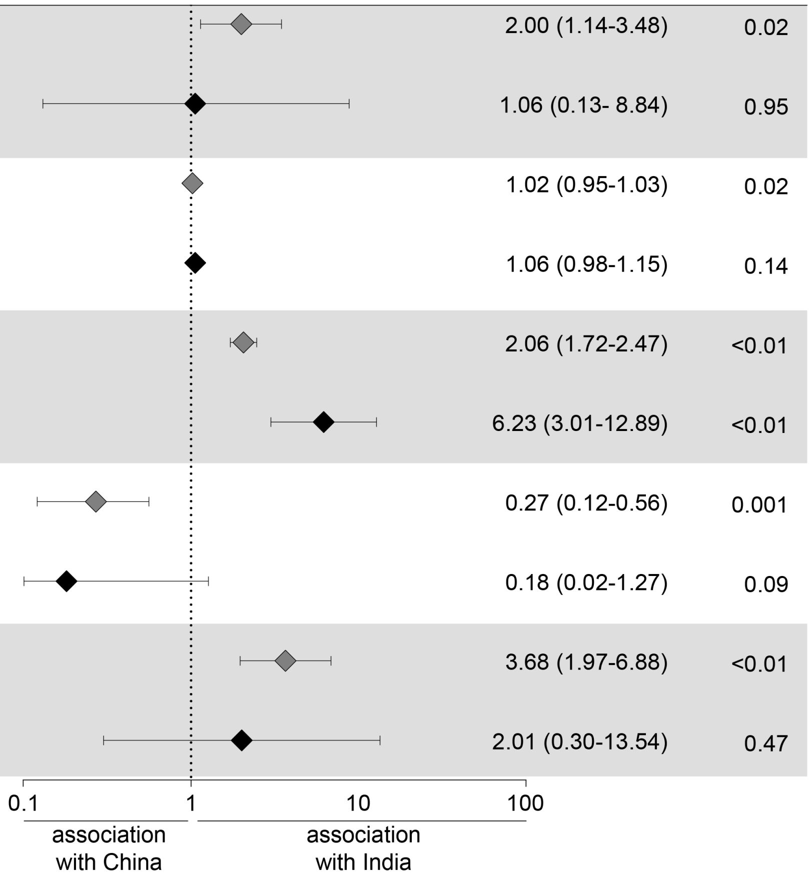

Figure S1. Factors associated with country of origin.
A multivariate regression model of MDP score values for all markers which displayed univariate p-value ≤ 0.2. Only variables who were statistically significant in univariate analyses are shown.

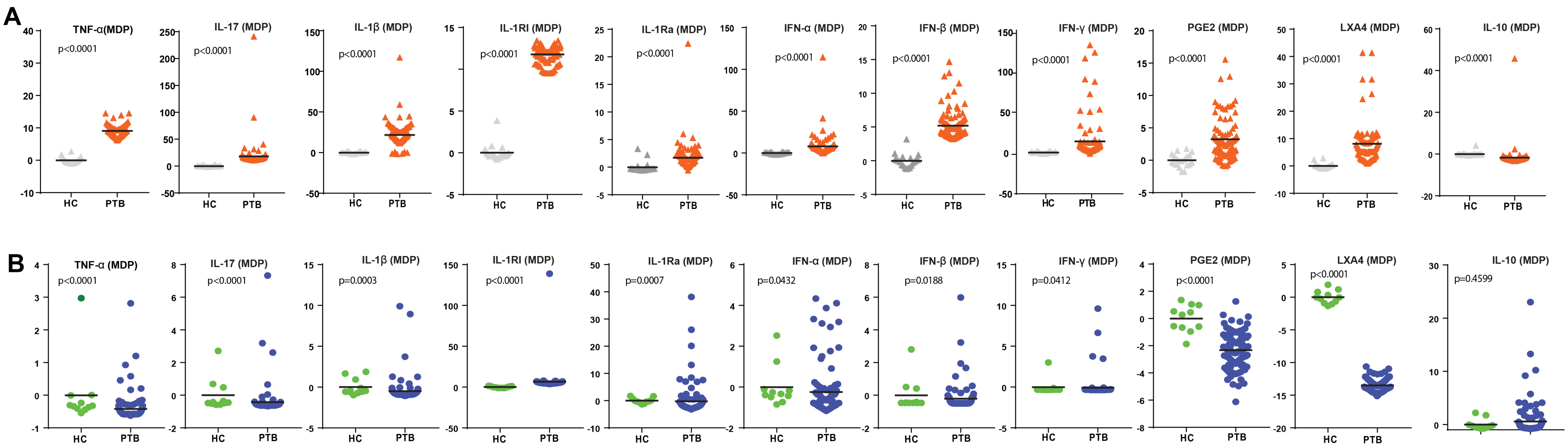

Figure S2. Differences in the inflammatory perturbation between India and China.
(A, B) Scatterplots of molecular degree of perturbation of indicated biomarkers of TB patients stratified per the group (India HC, n=20 and PTB, n=97; China HC, n=11 and PTB, n=100). Lines represent median values. Data were compared using the Mann–Whitney *U* test.

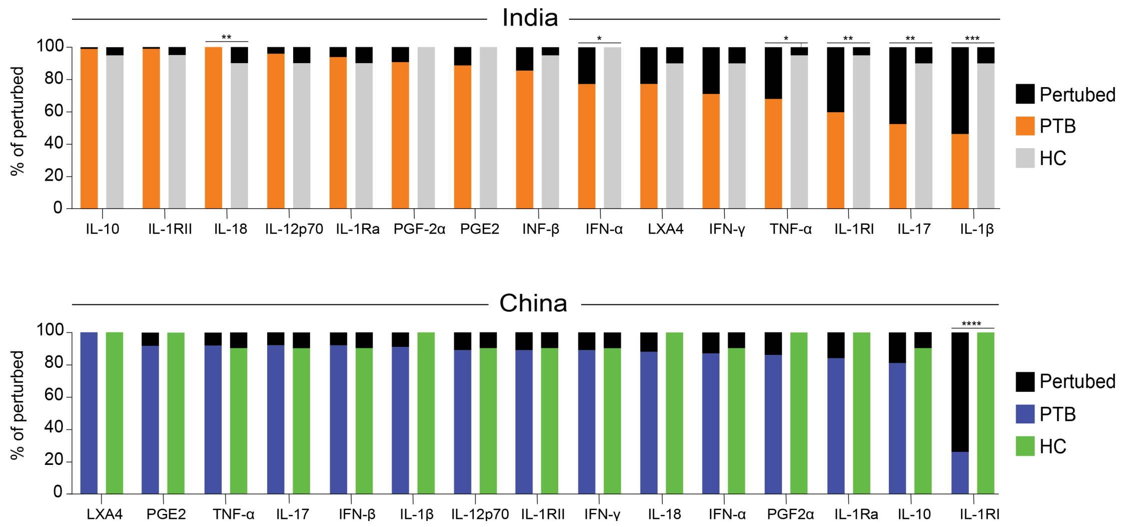

Figure S3. Frequency of individuals with molecular degree of perturbation by marker and country of origin.

MDP values were calculated for each markers and frequency of individuals who were molecularly perturbed were compared between PTB and HC groups from India (upper panel) or China (bottom panel) using the Fisher's exact test. *p<0.05, **p<0.01, ***p<0.001.

Parameter	Model	Odds Ratio (95% CI)	p-value
**Age**	**unadjusted**	1.03 (1.01-1-05)	0.01
	**adjusted**	1.06 (0.96-1.17)	0.25
**IL-10**	**unadjusted**	0.51 (0.39-0.67)	<0.01
	**adjusted**	0.85 (0.075-0.97)	<0.01
**IFN-α**	**unadjusted**	3.42 (2.44- 4.78)	<0.01
	**adjusted**	5.37 (2.04-14.17)	<0.01
**IFN-γ**	**unadjusted**	3.51 (2.48-4.96)	<0.01
	**ajusted**	2.57 (1.51-4.34)	<0.01
**AFB smear grade ≥ +2**	**unadjusted**	2.71 (1.31-5.63)	0.008
	**ajusted**	0.44 (0.01-24.84)	0.69
**Bilateral lung lesion**	**unajusted**	2.37 (1.14-4.94)	0.02
	**ajusted**	1.06 (0.02-68.24)	0.98

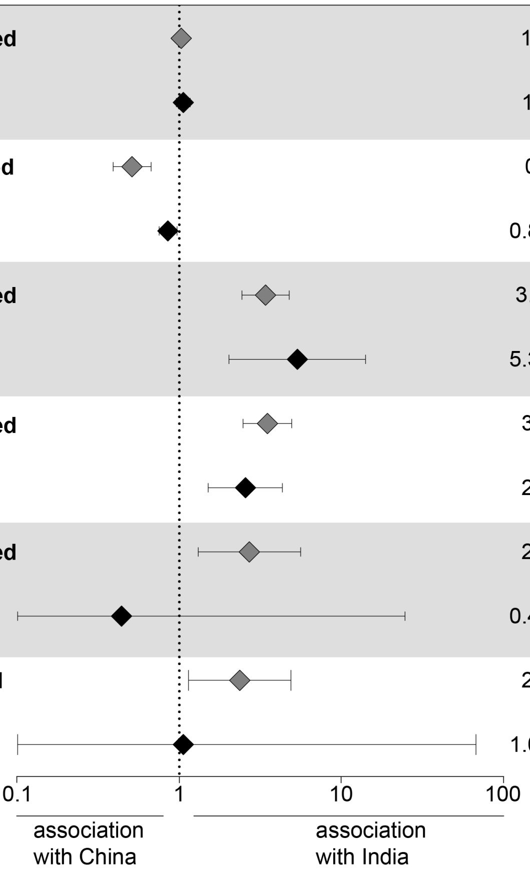

Figure S4. Factors associated with country of origin.
A multivariate regression model of MDP score values for all markers which displayed univariate p-value ≤ 0.2. Only variables who were statistically significant in univariate analyses are shown.

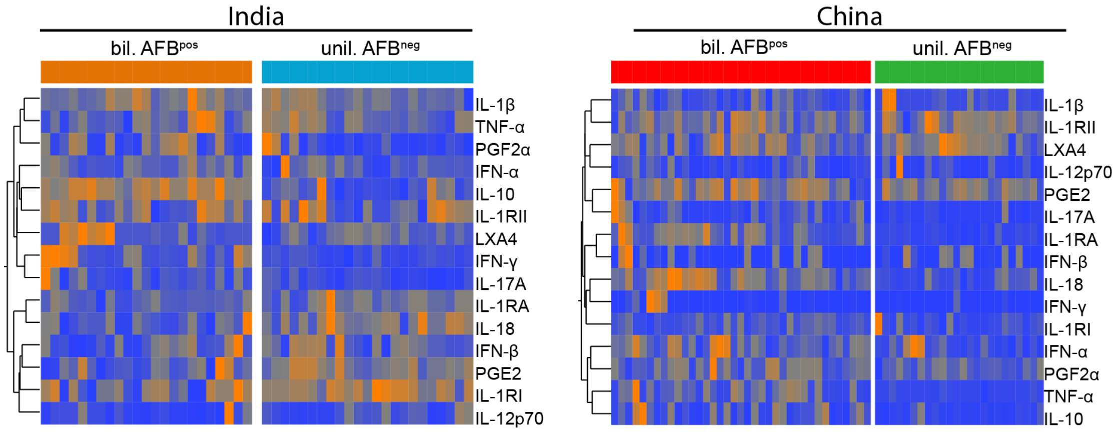

Figure S5. Molecular degree of perturbation in patients with distinct disease presentation profiles.

MDP score values for each biomarker were z-score normalized by country and a hierarchical cluster analysis (Ward's method with 100X bootstrap) was employed to illustrate the overall expression profile in PTB patients stratified per AFB smear status and lung disease extension (AFB^{neg} + unilateral disease and AFB^{pos} + bilateral disease). Orange color represents the highest values whereas blue denotes lowest values detected.
